# Gender diversity in adolescents with chronic liver disease: Presence and lived experience insights

**DOI:** 10.1002/jpn3.70389

**Published:** 2026-02-23

**Authors:** Katherine Wheatley, Jemma Day, Kate Annings, Marianne Samyn

**Affiliations:** ^1^ King's College Hospital (KCH) Young Adult Liver Service (YALS) London UK; ^2^ King's College London (KCL) London UK

**Keywords:** hepatology, mixed‐methods, thematic analysis, transgender, young adults

There is increasing awareness of transgender, gender‐queer, and non‐binary (TGQNB) adolescents in healthcare, representing at least 1% of 16–24‐year‐olds in England and Wales.^1^ Compared with cisgender (non‐TGQNB) peers, gender‐diverse individuals are disproportionately affected by chronic disease^2^ and face well‐documented disparities in healthcare access, outcomes, and engagement. These disparities are largely driven by systemic barriers,^3^ conceptualised in the gender minority stress framework (GMSF)^4^ as arising from external ‘distal’ stressors (e.g., misgendering, stigma), and internal ‘proximal’ stressors (e.g., concealment, anxiety about disclosure). Conversely, affirming clinical relationships, inclusive practices, and multidisciplinary (MDT) care with psychological support have been linked to safe identity disclosure and improved engagement.^3^

Evidence directly examining gender diversity in chronic liver disease is scarce, though broader adult literature indicates that gender minorities experience a higher burden of chronic physical health conditions, including liver‐related diseases.^2^ Young people with liver disease also show higher rates of neurodiversity,^5^ which is associated with increased TGQNB prevalance,^6^ raising questions about representation in this population. Safe disclosure of gender identity is essential to enable tailored holistic care, particularly given the liver's role in hormone metabolism and the potential implications of gender‐affirming therapies.^7^ Adolescents with liver disease are already more vulnerable to mental health difficulties,^8^ experiencing poorer outcomes than younger or older cohorts,^9^ often linked to psychosocial difficulties.^10^ Yet, how these factors intersect for TGQNB adolescents remains unexplored.

In this context, the present study explored the presence and lived experiences of TGQNB patients attending a specialised young adult liver service (YALS) which provides multidisciplinary care for young people aged 16–25 years, supporting transition from paediatric to adult services through integrating hepatology, nursing, psychology, and allied health input, with a focus on autonomy, engagement, and psychosocial wellbeing.

A concurrent nested mixed‐methods design was used. An anonymised online survey was offered to YALS patients between March and July 2024, via clinic posters and word‐of‐mouth. The survey captured demographic characteristics and gender identity, aligned to the England and Wales 2021 Census^1^ to enable comparison with national prevalence estimates (see File S1). No additional demographic or disease‐specific data were collected to preserve anonymity. Quantitative data were analysed descriptively, with proportions reported, and used to contextualise the qualitative findings.

A focus group with self‐selecting TGQNB patients, recruited by approaching individuals known to the service, explored experiences of identity disclosure, clinician interaction, and systemic barriers using a semi‐structured topic guide (see File S2). Data were analysed thematically,^11^ with initial coding informed by the Modified Gender Affirmation Framework^12^ to minimise researcher bias and subsequently mapped onto the GMSF^4^ to inform service improvement recommendations. Patient and Public Involvement (PPI) informed design, analysis, and recommendations.

Thirty‐two patients responded to the survey. Six (18.5%) reported a gender identity different from sex assigned at birth, compared with national estimates of 1% among people aged 16–24^1^ (Table 1).

**Table 1:** Gender identity characteristics of survey participants and 16–24 year olds from the 2021 Census for England and Wales.^8^

	Survey responses		Census (ages 16–24)
	*N*	%	%
Gender same as sex registered at birth	25	78.13	91.89
(Cis Woman)	(15)	(46.88)	(45.95)
(Cis Man)	(10)	(31.25)	(45.95)
Gender different from sex registered at birth	6	18.75	1.00
(Trans woman)	(2)	(6.25)	(0.15)
(Trans man)	(2)	(6.25)	(0.22)
(Non‐binary)	(1)	(3.13)	(0.26)
(Otherwise‐/Not‐specified)	(1)	(3.13)	(0.28)
Not answered	1	3.13	7.11

Ten patients were invited to participate in the focus group. Six expressed interest, five did not respond, one withdrew citing mistrust of the NHS, and one was unable to attend due to scheduling conflicts. Three ultimately provided informed consent and attended. The median age was 24 years, and participants had been seen in YALS for between 2 and 8 years. Further characteristics are not reported to protect confidentiality.

Three overarching themes and nine sub‐themes were identified and mapped onto the GMSF (Figure 1).

**Figure 1:**
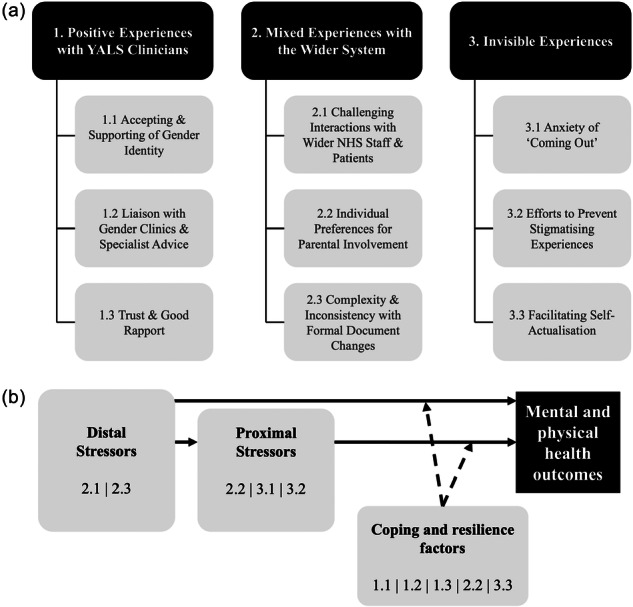
Summary of qualitative findings. (a) Themes and subthemes identified through thematic analysis. (b) Themes mapped onto the Gender Minority Stress Framework.^4^ YALS, young adult liver service.

YALS clinicians were perceived as affirming and respectful, using chosen names and responding to disclosures without altering demeanour or shifting focus away from liver care. Participants felt understood, valued, and not judged.

Not making it a big thing and reacting like a calm, normal way is definitely the best thing that helped me feel comfortable.

Clinicians' understanding of interactions between gender‐affirming treatments and CLD, alongside support with referral to gender specialist services, enabled personalised empowering discussions.

I haven't started testosterone because it can give you liver damage… I'll be having the gel because they can flush it out quickly if anything goes wrong.

Trust and rapport central to positive experiences with YALS clinicians were developed through developed through respectful communication, consistent affirmation, and clinician sensitivity, and enabled the first disclosures of gender identity in a medical setting.

That level of comfort helps us feel like ourselves rather than having to play a part.

Interactions with wider NHS staff and other patients were predominantly negative, with recurrent misgendering and judgement causing discomfort and anxiety.

It's just awkward… everyone looks at you like that's an awkward subject.

Two participants felt parental presence hindered disclosure and authentic self‐presentation, while for one provided emotional safety.

Having parents around the whole time was very awkward… I wanted to express this thing, but I'm wasn't sure.

Participants emphasised the importance of updating medical and legal records, yet described difficulties doing so, with errors contributing to missed appointments and distress.

I did change my name… but when [Clinician] came up to me, he called me by my dead name.

Disclosing gender identity was described as anxiety‐provoking, even in a supportive setting. Sources of anxiety included interoceptive factors (e.g., denial of one's own gender identity) and interpersonal concerns (e.g., clinicians' responses).

You see us in our most vulnerable states physically, we're now opening up with our most vulnerable state emotionally.

Participants modified appearance or withheld their chosen name to protect themselves from discrimination.

If you're open to a couple of the healthcare members, how will others react, like other patients, receptionists or cleaners?

Despite challenges, YALS was experienced as a space that fostered confidence and empowerment and supported informed decision‐making about liver care.

I'm a person first and then I am trans second… The only thing that matters is whatever's relevant to my health.

In this study, 18.5% of young people with CLD identified as TGQNB, compared with national estimates of approximately 1% among 16–24‐year‐olds.^1^ Although based on a small, self‐selected sample, this observation highlights that clinicians in adolescent liver services are likely to encounter TGQNB patients and should be prepared to support their needs. While disease‐related factors were not examined, this raises questions about how gender diversity intersects mental health and neurodevelopmental vulnerabilities in this population.^5^, ^6^

Qualitative findings identified factors that facilitated and hindered safe disclosure and engagement. Choice regarding parental involvement, opportunities to discuss identities beyond liver disease, and clinician competence in discussing gender identity all supported disclosure. Following disclosure, affirming clinical relationships (characterised by consistent demeanour, normalising responses, and consistent focus on liver care including practical support if considering gender‐affirming hormone therapy) were central to trust and engagement. Conversely, systemic barriers such as inflexible documentation, inconsistent use of names/pronouns, limited staff awareness, and lived or anticipated discrimination undermined care experiences and delayed disclosure.

These findings mirror broader healthcare literature demonstrating that affirming encounters support disclosure and engagement, while structural inequalities negatively impact TGQNB populations.^3^, ^4^, ^12^ For clinicians, this underscores the importance of openness, avoiding assumptions, regularly checking preferred names/pronouns in electronic records, and allowing young people to disclose aspects of identity on their own terms. This approach also supports informed, shared decision‐making for those considering gender‐affirming hormone therapy, where the liver's role in hormone metabolism requires careful consideration in the context of liver disease.^7^, ^10^ At service‐level, visible markers of inclusivity (e.g., pronoun badges), staff training, and better integration of preferred name/pronoun fields within electronic systems may help create safer environments for disclosure and sustained engagement.

This study was strengthened by meaningful PPI throughout, supporting relevance and rigour. Limitations include opportunity and volunteer sampling, small sample sizes, and the anonymous survey design, which may have introduced bias, limited representativeness (e.g., underrepresentation of patients mistrustful of healthcare), and precluded examination of disease‐related factors. Consequently, it remains unclear whether the higher prevalence observed reflects over‐reporting or a true association between TGQNB identities and liver disease. Replication in larger, multicentre cohorts is needed to clarify prevalence and explore underlying mechanisms, including the potential roles of disease‐related factors such as age at diagnosis, neurodiversity and hormone profiles.

This study highlights that clinicians are likely to encounter TGQNB adolescents in liver services, with almost on in five participants in the current sample identifying as such. Affirming clinical relationships supported disclosure, trust, and engagement; while systemic barriers including inflexible documentation, inconsistent pronoun use, and limited staff awareness undermined care experiences. These findings emphasise the importance of inclusive practices and service adaptations to support safe disclosure, alongside the need for further research in larger cohorts to clarify prevalence and inform strategies to reduce systemic barriers. Embedding identity‐informed practices into multidisciplinary hepatology services may improve equity, engagement, and outcomes for adolescents with liver disease.

## CONFLICT OF INTEREST STATEMENT

The authors declare no conflicts of interest.

## ETHICS STATEMENT

This project was approved by King's College Hospital Trust research committee (reference CH166).

## Supporting information

Supporting information.

Supporting information.
