# Supplementary material for: Gender diversity in adolescents with chronic liver disease: Presence and lived experience insights
Source: J Pediatr Gastroenterol Nutr. 2026 Feb 23;82(5):1284–7. doi: 10.1002/jpn3.70389 (PMC13150789; doi:10.1002/jpn3.70389)
Supplement: Supplementary file 1 — Supporting information. [file JPN3-82-1284-s002.docx]

| **Supplementary Materials 1**  *Online Survey Questions* | |
| --- | --- |
| Question | Response Type |
| What is your sex? A question about gender identity will follow later on in the questionnaire | Closed-ended (Yes / No) |
| Is the gender you identify with the same as your sex registered at birth? | Closed-ended (Yes / No) |
| If you answered No, what is your gender identity? | Open-ended text |
