# Supplementary material for: Gender diversity in adolescents with chronic liver disease: Presence and lived experience insights
Source: J Pediatr Gastroenterol Nutr. 2026 Feb 23;82(5):1284–7. doi: 10.1002/jpn3.70389 (PMC13150789; doi:10.1002/jpn3.70389)
Supplement: Supplementary file 2 — Supporting information. [file JPN3-82-1284-s001.docx]

| **Supplementary Materials 2**  *Focus Group Interview Schedule* | |
| --- | --- |
| Key Questions | Prompts |
| Have you ever had a conversation about your gender identity with any of the staff in the young adults liver service or while you’ve been an inpatient? | - Who initiated the conversation  - What prompted the conversation |
| What helped, or didn’t help you share your gender identity with the service | - Barriers to sharing  - Facilitators to sharing |
| What recommendations would you like to give the young adults liver service to help them feel better equipped to support people who are transgender and non-binary and gender | - Negative experiences  - Positive experiences  - Practical suggestions |
| Is there anything else that you would like to share about your experience of the young adults liver service at King’s College Hospital as a transgender, non-binary, or gender-queer young adult? |  |
